# LncRNA MIR100HG promotes cell proliferation in triple-negative breast cancer through triplex formation with p27 loci

**DOI:** 10.1038/s41419-018-0869-2

**Published:** 2018-07-24

**Authors:** Shaowei Wang, Hao Ke, Honglei Zhang, Yujie Ma, Lei Ao, Li Zou, Qin Yang, Hao Zhu, Jianyun Nie, Chunlian Wu, Baowei Jiao

**Affiliations:** 1 Key Laboratory of Southwest China Wildlife Resources Conservation, China West Normal University, Ministry of Education, Nanchong, 637009 China; 2 State Key Laboratory of Genetic Resources and Evolution, Kunming Institute of Zoology, Chinese Academy of Sciences, Kunming, 650223 China; 3 Kunming College of Life Science, University of Chinese Academy of Sciences, Kunming, 650223 China; 4 Kunming Angel Women’s and Children’s Hospital, Kunming, 650032 China; 5 Bioinformatics Section, School of Basic Medical Sciences, Southern Medical University, Guangzhou, 510515 China; 6 Department of Breast Cancer, Yunnan Cancer Center, Third Affiliated Hospital of Kunming Medical University, Kunming, 650118 China; 7 Center for Excellence in Animal Evolution and Genetics, Chinese Academy of Sciences, Kunming, 650223 China

## Abstract

Triple-negative breast cancer (TNBC) exhibits poor prognosis, with high metastasis and low survival. Long non-coding RNAs (lncRNAs) play critical roles in tumor progression. Here, we identified lncRNA MIR100HG as a pro-oncogene for TNBC progression. Knockdown of MIR100HG decreased cell proliferation and induced cell arrest in the G1 phase, whereas overexpression of MIR100HG significantly increased cell proliferation. Furthermore, MIR100HG regulated the p27 gene to control the cell cycle, and subsequently impacted the progression of TNBC. In analyzing its underlying mechanism, bioinformatics prediction and experimental data demonstrated that MIR100HG participated in the formation of RNA–DNA triplex structures. MIR100HG in The Cancer Genome Atlas (TCGA) and breast cancer cell lines showed higher expression in TNBC than in other tumor types with poor prognosis. In conclusion, our data indicated a novel working pattern of lncRNA in TNBC progression, which may be a potential therapeutic target in such cancers.

## Introduction

Breast cancer is a molecularly-heterogeneous disease and can be classified into four molecular subtypes, including luminal A, luminal B, human epidermal growth factor receptor type 2 (HER2) positive, and triple-negative breast cancer (TNBC)^1,2^. Among them, TNBC, which demonstrates a lack of progesterone receptor, estrogen receptor, and HER2 expression by immunohistochemistry, is a highly invasive subtype comprising 10%−20% of all breast cancer cases^3,4^. TNBC can exhibit high invasion, distant metastasis, high recurrence risk, poor prognosis, and low survival^4^. Unlike other subtypes, endocrine therapy or HER2-targeted therapies are relatively ineffective in TNBC^5,6^. At present, although great endeavors have been made in clinical treatment strategies, patient survival has not improved remarkably. Thus, exploration of the molecular mechanisms and development of more effective therapeutic strategies are critical.

LncRNA molecules are longer than 200 nucleotides in length, but lack obvious open reading frames^7,8^. Their transcripts can be transcribed by RNA polymerase II and exhibit typical mRNA-like features, such as 5′caps^9^. A variety of lncRNAs have been identified as evolutionarily non-conserved^10,11^. LncRNAs can be divided into intronic, bidirectional, intergenic, sense, and antisense according to their position in the genome^7^. Growing evidence suggests the involvement of lncRNAs in important cellular processes, including epigenetic regulation^12,13^, transcriptional regulation^14^, and chromosome inactivation^15^. Moreover, lncRNAs play a crucial part in tumor biology^16,17^. A number of novel cancer-related lncRNAs have been successfully identified by high-throughput sequencing, with lncRNAs found to be abnormally expressed in many cancers^18,19^.

Corresponding to the powerful roles of lncRNAs, research has reported on various working mechanisms, including interactions with DNA, RNA, and proteins^20^. Many previous studies have focused on the interactions between lncRNAs and their protein partners in the regulation of gene expression^21–23^. However, little attention has been paid to the formation of RNA–DNA triplex structures. LncRNAs can recognize and bind to specific double-stranded DNA sequences to form RNA–DNA triplex structures. Under physiological conditions, triplex-forming oligonucleotides (TFOs) can bind to the major groove of the targeted duplex through sequence-specific recognition of the polypurine sequence^24–26^. RNA–DNA triplex structures are also reported to be involved in cancer, targeting specific sequences in DNA and regulating gene expression at the transcriptional level^27,28^. Research has demonstrated that synthetic TFOs can directly bind to the Ets2 promoter sequence, with Ets2-TFO able to suppress the expression of endogenous genes and the activity of the Ets2 promoter in prostate cancer^29^, suggesting that this RNA–DNA triplex structure may play an important role in tumor progression. However, whether endogenous lncRNAs participate in the formation of RNA–DNA triplex structures in cancer progression remains unknown.

MIR100HG is a microRNA host gene located on chromosome 11q24.1, and encodes three microRNAs in its introns, including mir-100, mir-125b-1, and let-7a-2^30^. Several studies have reported on the role of this lncRNA in tumor progression^30–34^. MIR100HG is highly expressed in acute megakaryoblastic leukemia (AMKL), with its knockdown shown to decrease AMKL cell proliferation and viability^31^, High expression of MIR100HG is also related to poor prognosis in early-stage cervical cancer^34^, and MIR100HG, miR-125b, and miR-100 are reportedly overexpressed in cetuximab-resistant colorectal cancer^30^. These previous studies have primarily focused on the relationship between MIR100HG and its host microRNAs. Here, however, we showed that MIR100HG was more highly expressed in poor prognosis TNBC compared with other breast cancer subtypes. Loss-of-function assays demonstrated that reduced MIR100HG inhibited cell growth and induced cell arrest in the G1 phase. These results were confirmed by gain-of-function assays. High-throughput sequencing and further experiments showed that MIR100HG negatively regulated p27 expression, and hence increased cell proliferation in TNBC. Of note, we found that MIR100HG regulated p27 through the formation of an RNA–DNA triplex structure, thus providing a new avenue for lncRNA studies.

## Results

### MIR100HG promotes proliferation in TNBC cells

**Fig. 1:**
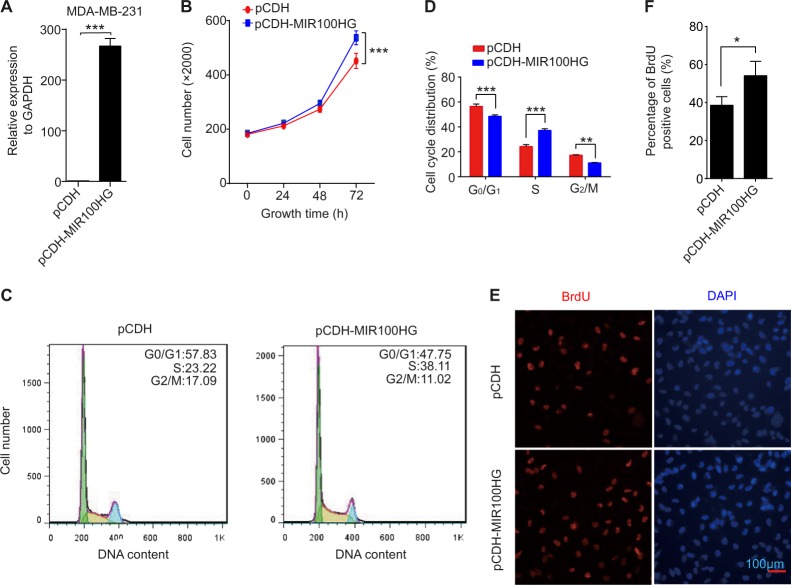
Upregulation of MIR100HG increased TNBC cell proliferation. **a** qPCR analysis of MIR100HG expression in MDA-MB-231 cells transfected with pCDH-MIR100HG or empty pCDH vector. Transcript levels were normalized to GAPDH expression. **b** MTS assay determined the proliferation of pCDH-MIR100HG-transfected MDA-MB-231 cells. **c**, **d** FACS determined the relative cell numbers in each cell-cycle phase after propidium iodide staining of MIR100HG-overexpressed MDA-MB-231 cells. **e**, **f** BrdU staining of cells with MIR100HG overexpression. The data represent three independent experiments. **P < 0.01, ***P < 0.001 by one-way ANOVA

To explore the roles of MIR100HG in TNBC progression, we first overexpressed MIR100HG by transfecting the pCDH plasmid containing full-length MIR100HG in TNBC MDA-MB-231 cells (Fig. 1a). Quantitative PCR (qPCR) showed a nearly 300-fold increase in the expression level of MIR100HG, indicating successful overexpression. MTS assay demonstrated that overexpression of MIR100HG significantly increased cell growth, suggesting that overexpression promoted TNBC cell proliferation (Fig. 1b). Flow cytometry indicated that overexpression of MIR100HG in MDA-MB-231 cells increased the percentage of cells in the S phase compared with pCDH cells (Fig. 1c, d). Furthermore, significantly higher BrdU staining also showed an increase in the S phase after overexpressing MIR100HG, suggesting the promotion of TNBC DNA replication (Fig. 1e, f). To identify whether MIR100HG could be translated into a protein, we predicted its translation ability using the Coding Potential Calculator^35^. Results indicated that MIR100HG did not have the potential or ability to translate into a protein (data not shown).

**Fig. 2:**
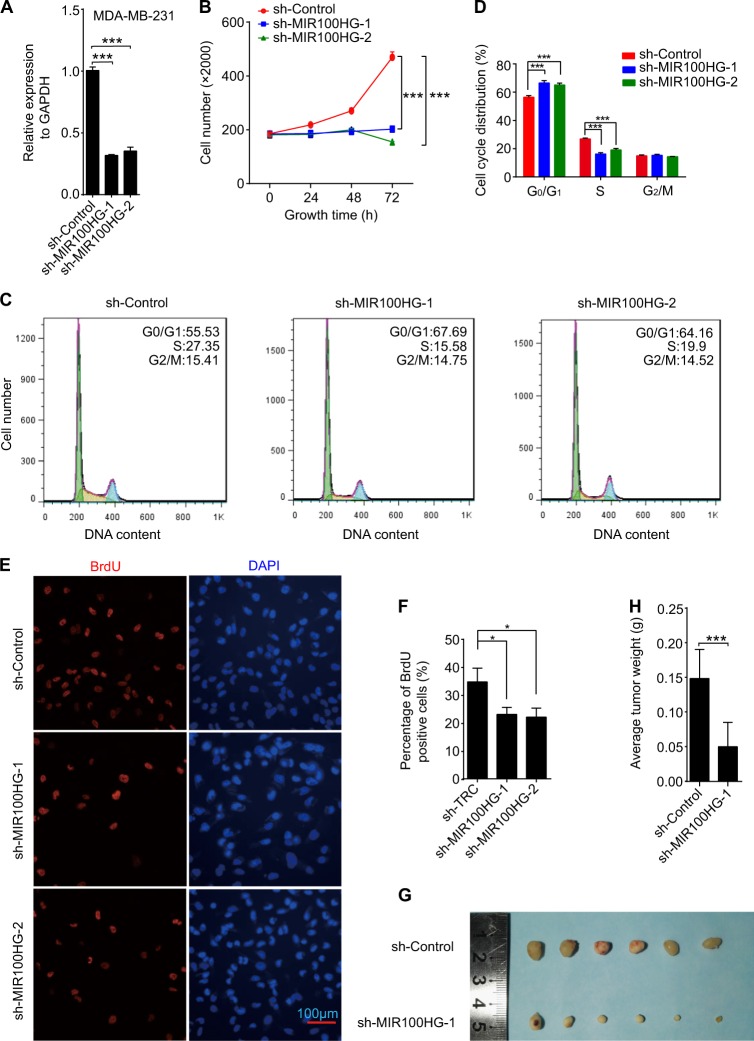
Downregulation of MIR100HG decreased TNBC cell proliferation. **a** qPCR analysis of the downregulation of MIR100HG by two shRNAs in MDA-MB-231 cells. Transcript levels of MIR100HG were normalized to GAPDH expression. **b** MTS assay determined the proliferation of sh-MIR100HG-transfected MDA-MB-231 cells. **c**, **d** FACS determined the relative cell numbers in each cell-cycle phase after propidium iodide staining of MIR100HG-downregulated MDA-MB-231 cells. (**e**-**f**) BrdU staining assay of cells following knockdown of MIR100HG. **g**, **h** Photographs of tumors (**g**) and tumor weights (**h**) derived from MDA-MB-231 cells eight weeks after xenograft. The data represent three independent experiments (**a**–**f**). ***P < 0.001 by one-way ANOVA

In addition to gain-of-function assays, we also performed loss-of-function assays. Two MIR100HG shRNAs were designed to exclude off-target effects, with shRNA lentiviruses then used to infect the two TNBC cell lines, (i.e., MDA-MB-231 and BT549 cells) (Fig. 2a and Supplementary Figure S1A). Consistent with the overexpression experiments, our results showed that knockdown of MIR100HG significantly reduced MDA-MB-231 cell proliferation (Fig. 2b). These results were also observed in BT549 cells (Supplementary Figure S1B). Flow cytometry showed that reduced MIR100HG expression led to cell arrest in the G1 phase (Fig. 2c, d). BrdU staining also showed the suppression of proliferation by knockdown of MIR100HG in MDA-MB-231 cells (Fig. 2e, f). To confirm the MIR100HG results, we performed a xenograft assay on tumor growth using MDA-MB-231 cells, which showed that stable knockdown of MIR100HG significantly impaired tumor growth (Fig. 2g, h).

The above results suggest that MIR100HG promotes cell proliferation in TNBC cell lines.

### MIR100HG leads to cell cycle arrest at G1/S via p27 regulation

**Fig. 3:**
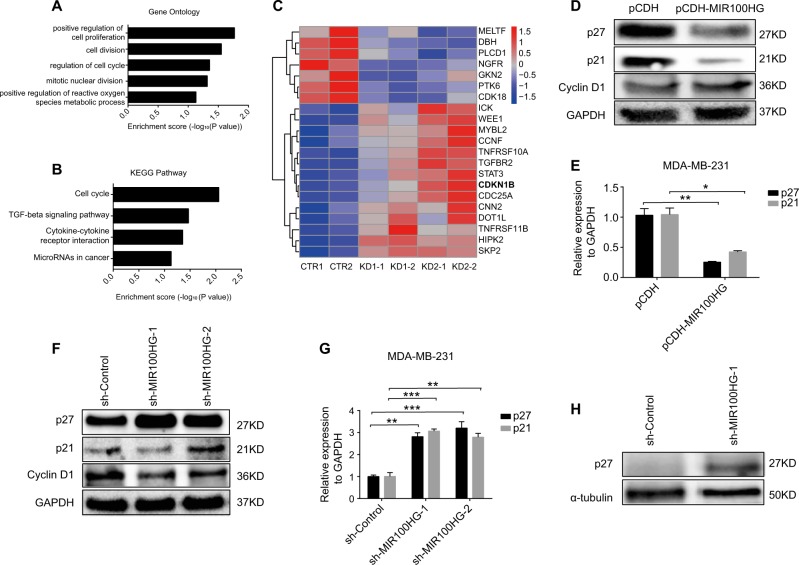
MIR100HG induced cell cycle arrest at G1/S via p21/p27 regulation. **a**, **b** Gene ontology analysis (**a**) and KEGG pathway analysis (**b**) for RNA-seq data. **c** Heatmap of 21 coding genes related to the cell cycle with significantly altered expression following knockdown of MIR100HG in MDA-MB-231 cells. KD1-1 and KD1-2 are two biological replicates of sh-MIR100HG-1; KD2-1 and KD2-2 are two biological replicates of sh-MIR100HG-2. **d**, **e** Effect of pCDH-MIR100HG on expressions of p27, p21, and cyclin D1 at the protein (**d**) and RNA (**e**) levels. **f**, **g** Effect of sh-MIR100HG on expressions of p27, p21, and cyclin D1 at the protein (**f**) and RNA (**g**) levels. **h** Detection of p27 expression by western blot analysis using xenograft assay samples. The data represent three independent experiments (**d**–**g**). *P *<* 0.05, **P < 0.01, ***P < 0.001 by one-way ANOVA

To reveal how MIR100HG affects TNBC progression, we performed RNA-seq after knockdown of MIR100HG in MDA-MB-231 cells. Results showed that 387 cancer-related genes exhibited significant changes in expression (Log2FC > 2 or Log2FC < −2 and FDR < = 0.05) between the MIR100HG shRNA groups and their controls (data not shown). Gene ontology analysis demonstrated that these genes are involved in cell cycle, cell division, and cell proliferation (Fig. 3a). KEGG pathway analysis also showed high enrichment of pathways involved in the cell cycle (Fig. 3b). Cell cycle-related genes were markedly changed between the MIR100HG knockdown and control groups, and included CDK18, WEE1, CCNF, CDKN1B, and CDC25A (Fig. 3c). CDKN1B, which encodes the p27 protein, is a tumor suppressor that regulates cell cycle proliferation and, as a cyclin-dependent kinase inhibitor, controls cell cycle progression at the G1 phase^36,37^. Thus, we reasoned that MIR100HG could affect TNBC progression through regulation of p27 expression. At the same time, p21 and cyclin D1 were also investigated due to their important roles in the G1 phase. The expression of p21 and p27 decreased at both the protein and mRNA level (Fig. 3d, e), and the expression of cyclin D1 increased with MIR100HG overexpression (Fig. 3d). Consistent with the overexpression experiments, knockdown of MIR100HG increased p21 and p27 at the protein and RNA levels (Fig. 3f, g). In contrast, the expression of cyclin D1 was decreased (Fig. 3f). The tissue of tumor-bearing mice was used to detect the expression of p27, with knockdown of MIR100HG found to significantly increase the protein expression level of p27 (Fig. 3h). Ectopic expression of MIR100HG did not change the levels of neighbor genes such as *BLID*, *UBASH3B*, and *SORL1* or of microRNAs such as miR-100, miR125b-1, and let-7a-2 in either MDA-MB-231 or BT549 cells (Supplementary Figure S1C-F), suggesting that the effect of MIR100HG was not through hosted microRNAs in TNBC cells.

### MIR100HG and p27 form a triplex structure

To investigate how MIR100HG regulates p27 expression, we first employed nuclear cytoplasmic separation to identify the subcellular localization of MIR100HG. We used *GAPDH* and *ACTIN*, which are mainly located in the cytoplasm, as negative controls and *MALAT1*, which is located in the nucleus, as a positive control to validate the qPCR assay (Supplementary Figure S2A). Our results indicated that MIR100HG was mainly located in the nucleus, providing the possibility of interaction with genomic DNA.

**Fig. 4:**
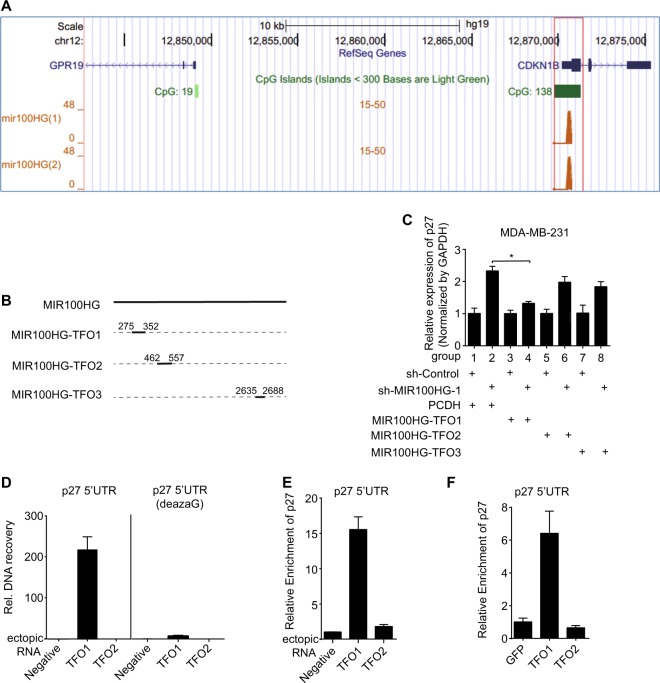
MIR100HG and p27 formed an RNA–DNA triplex structure. **a** Triplex structure prediction of MR100HG and p27 using LongTarget. **b** Vector constructions of pCDH-MIR100HG-TFO1, pCDH-MIR100HG-TFO2, and pCDH-MIR100HG-TFO3. **c** P27 levels by MIR100HG knockdown with/without overexpression of pCDH-MIR100HG-TFO1, pCDH-MIR100HG-TFO2, and pCDH-MIR100HG-TFO3. **d** MIR100HG physically interacted with p27 in vitro. P27 DNA duplex fragment ( + 141/ + 469) with normal dGTP (left) or with 7-deaza-dGTP (right) were pulled down by biotinylated TFO1,TFO2 probes, and thereafter quantified by qPCR. **e** MIR100HG interacted with p27 in nuclei. Biotinylated TFO1,TFO2 was incubated with nuclei and then bound to streptavidin beads. RNA-associated DNA was quantified by qPCR. **f** MIR100HG interacted with p27 in vivo. Biotinylated TFO1,TFO2 was incubated with chromatin and streptavidin beads. RNA-associated DNA was quantified by qPCR. The data represent three independent experiments (**c**–**f**). *P < 0.05 by one-way ANOVA

Recent studies have shown that nuclear RNA can bind to specific genomic loci to form RNA–DNA triplex structures, which can, in turn, regulate gene transcription^38,39^. Since the formation of ncRNA–DNA triplexes follows specific base-pairing rules^26,40^, the binding motifs (triplex-forming oligonucleotides, TFOs) in ncRNAs and binding sites (triplex target sites, TTS) in the genome can be effectively predicted computationally. We used the LongTarget program to predict MIR100HG TFOs and their TTSs in the p27 region^41^. Through bioinformatics prediction, we found three TFOs in MIR100HG that specifically bound to p27 to form RNA–DNA triplex structures (Fig. 4a), which were located at 275–352 nt, 462–557 nt, and 2635–2688 nt of MIR100HG, respectively. Therefore, we inserted these sequences into pCDH vectors, named pCDH-MIR100HG-TFO1, pCDH-MIR100HG-TFO2, and pCDH-MIR100HG-TFO3, respectively (Fig. 4b). Following MIR100HG knockdown, the expression of p27 increased (group 2 in Fig. 4c), consistent with our earlier results (Fig. 3f–h). Overexpressed TFO1, but not TFO2 and TFO3, significantly abated the increase in p27 expression caused by MIR100HG knockdown (group 4 in Fig. 4c), suggesting that TFO1 can rescue the effects triggered by MIR100HG loss. To further confirm their role, we performed concomitant overexpression of TFOs (TFO1 + TFO2, TFO1 + TFO3, and TFO1 + TFO2 + TFO3) to determine possible synergistic effects among TFO1, TFO2, and TFO3 in the regulation of p27 expression. As shown in Supplementary Figure S2B, overexpressed TFO1 (group 4 in Supplementary Figure S2B), TFO1 + TFO2 (group 6 in Supplementary Figure S2B), and TFO1 + TFO3 (group 8 in Supplementary Figure S2B) partially abated the increase in p27 expression caused by loss of MIR100HG. However, additional overexpression of TFO2 (TFO1 + TFO2) and TFO3 (TFO1 + TFO3) weakened, rather than enhanced, the effects of TFO1 on p27 expression. Moreover, overexpression of TFO1 together with TFO2 and TFO3 (TFO1 + TFO2 + TFO3, group 10 in Supplementary Figure S2B) completely abolished the TFO1-related effects on p27 expression in cells expressing sh-MIR100HG. These results imply that although TFO2 and TFO3 cannot change p27 expression levels, they may compete with TFO1 in binding to p27 gene loci, thereby negatively affecting the TFO1-dependent regulation of p27 expression. Therefore, TFO1, TFO2, and TFO3 do not appear to work synergistically, with TFO1 of MIR100HG found to be the functional binding site on p27 loci.

To determine whether TFO1 can bind to the DNA duplex, we performed an in vitro assay^38^ by amplifying the p27 DNA double-strand fragment at 5′UTR ( + 141/ + 469), known as triplex target DNA sites (TTSs), to bind to the TFO1 sequence (Fig. 4a) and thereafter co-incubate with biotin-labeled TFO1 probe for enrichment tests (qPCR). Results demonstrated that the p27 TTS sequence was significantly enriched at TFO1, but not at TFO2 (Fig. 4d, left). Moreover, we mutated the TTS fragment by amplifying the DNA duplex with 7-deaza-dGTP, which decreases the binding affinity of the DNA duplex to RNA^38^, and showed clearly weakened enrichment (Fig. 4d, right). Using the same method, we found that biotin-labeled TFO1 can bind to p27 in the nucleus (Fig. 4e). Therefore, we concluded that MIR100HG can bind to p27 through the RNA–DNA triplex. To prove that MIR100HG binds to p27 in vivo, we performed CHIRP assay by incubating biotin-labeled TFO1 with TNBC cell lysate. The qPCR assay indicated significant enrichment of p27 on TFO1, but not on TFO2 (Fig. 4f).

Taken together, our data demonstrated that MIR100HG, through the TFO1 sequence, binds to p27 gene loci to form RNA–DNA triplex structures, which subsequently regulate the expression of p27 (Supplementary Figure S2C).

### MIR100HG is highly expressed in poor survival TNBC

**Fig. 5:**
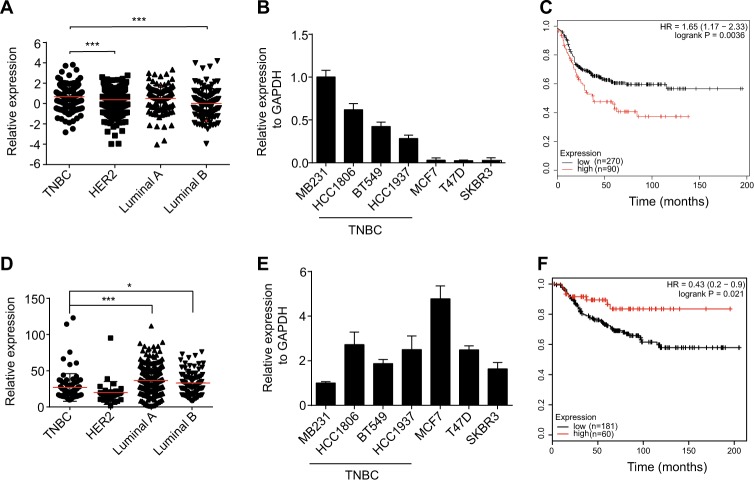
MIR100HG exhibited high expression in TNBC with poor survival. **a** MIR100HG expression in four molecular subtypes of breast cancer from the TCGA database. **b** qPCR analysis of MIR100HG expression in breast cancer cell lines. Transcript levels were normalized to GAPDH expression. **c** Kaplan–Meier curves for overall survival rate of 360 patients with breast cancer by MIR100HG expression in tumors (*P* = 0.0036).**d** P27 expression in four molecular subtypes of breast cancer from the TCGA database. **e** qPCR analysis of p27 expression in breast cancer cell lines. Transcript levels were normalized to GAPDH expression. **f** Kaplan–Meier curves for overall survival rate of 241 patients with breast cancer by p27 expression in tumors (*P* = 0.021). *P *<* 0.05, ***P < 0.001 by one-way ANOVA

In analyzing expression profiles of breast cancer subtypes based on The Cancer Genome Atlas (TCGA) database, MIR100HG showed significantly higher expression in TNBC than in other subtypes of breast cancer (Fig. 5a). We also detected MIR100HG in various breast cancer cell lines and found higher expression in TNBC cells lines than in other cell types (Fig. 5b). Moreover, Kaplan–Meier survival analysis from the KM plot data of TNBC patients (Fig. 5c), but not of other breast cancer subtypes (Supplementary Figure S1G-I), showed that higher expression of MIR100HG was associated with lower survival rates. From the TCGA database of the same cohorts with MIR100HG, we found that p27 was significantly lower in TNBC compared with other breast cancer subtypes (Fig. 5d). Furthermore, p27 in various breast cancer cell lines detected by qPCR also showed lower expression in TNBC cell lines than in other cell types (Fig. 5e). Survival analysis of basal subtypes demonstrated better survival probability in patients with higher p27 expression (Fig. 5f).

## Discussion

MIR100HG has been linked to the progression of a few cancers^30,31,34^. Previous studies have focused on its interaction with miRNAs, which share common gene loci with MIR100HG. As a miRNA host gene, MIR100HG can silence miR-100 expression through epigenetic modification in breast cancer^32^. MIR100HG, miR-125b, and miR-100 also contribute in coordination to cetuximab resistance in colorectal cancer^30^. Furthermore, MIR100HG has been found to function independently of the host miR-125b in human neuroblastoma^42^. In the current study, we found that MIR100HG did not function through miR-100, miR-125b, or let-7a-2 (Supplementary Figure S1D, F). We functionally characterized MIR100HG in TNBC and determined that it formed an RNA–DNA triplex structure with p27 gene loci.

Recent studies have demonstrated the involvement of lncRNAs in the formation of RNA–DNA triplex structures^38,39,43,44^. Antisense lncRNA PARTICLE, which is located within MAT2A gene loci, can be transcribed from the MAT2A promoter when cells are exposed to low doses of radiation. PARTICLE can suppress MAT2A expression via formation of a triple helix and interaction with PRC2^43^. Another study reported that an RNA–DNA triplex structure in the promoter regions of Foxf1 and Pitx2 can act as an anchor to recruit PRC2, which regulates tissue differentiation^44^. Accumulating evidence shows that RNA–DNA triplex structures play critical roles in lncRNA function^38,39^. For example, they can act as anchors to recruit DNA methylation enzymes or histone-modifying proteins to specific sites in chromatin^38,39^, which can, in turn, regulate gene transcription. TFOs serve as transcriptional suppressors and can downregulate gene expression in cancer^29,45–47^. For example, TFOs directed to a particular sequence in the Ets2 promoter can repress transcription of this gene, thereby inducing growth inhibition and apoptosis in prostate cancer^46^. Myc-targeted TFOs can downregulate c-myc expression and induce growth arrest and death of tumor cells^47^. Moreover, triplex formation in the c-MYC gene can induce anti-proliferative activity in breast cancer^48,49^, leukemia^50^, and ovarian and cervical carcinomas^51^. However, there are few reports on the involvement of lncRNAs in the formation of triplex structures in cancer. Triplex structure formation provides a universal mechanism for targeting specific DNA sequences by long intergenic noncoding RNAs to chromatin modifiers in the genome^52^. In our study, we found that MIR100HG bound to p27 and formed a triplex structure to regulate cell proliferation in TNBC. In general, lncRNAs can interact with EZH2 to repress p27 through histone methylation (H3K27me3) on p27 promoters^53^ or interact with transcription factors, which directly bind and activate the p27 promoter in tumors^54^. We found a new mechanism, whereby MIR100HG formed a triplex structure with p27, which might recruit epigenetically-modified proteins to directly bind and activate the p27 promoter to regulate cell proliferation in TNBC (Supplementary Figure S2C, D).

It has been well reported that both p21 and p27 act as cyclin-dependent kinase inhibitors^55^, and cyclin D1 is required for cell cycle progression in G1^56^. Our results showed that the expression of p21 and p27 decreased at both the protein and mRNA levels, and the expression of cyclin D1 increased with MIR100HG overexpression (Fig. 3d, e). Consistent with the overexpression experiments, knockdown of MIR100HG increased p21 and p27 at the protein and RNA levels. In contrast, the expression of cyclin D1 was decreased (Fig. 3f, g). p21, p27, and cyclin D1 are G1/S checkpoint cell cycle regulatory proteins, with p21 and p27 acting on the CDK4/6-cyclin D1 complex, thereby preventing cell cycle progression in the G1 phase. Both p21 and p27 act as cell cycle inhibitors, deterring G1 phase progression, whereas cyclin D1 promotes G1/S transition. These results were consistent with our findings on MIR100HG-dependent phenotypes (Figs. 1, 2). To determine the potential of MIR100HG to form DNA-RNA triplex structures and thereby regulate targeted gene expression, we performed bioinformatics prediction and found that MIR100HG could specifically form RNA–DNA triplex structures with the gene loci of p27 (CDKN1B) (Fig. 4a), but not with that of p21 or cyclin D1. Subsequently, we focused on the characterization of the MIR100HG-CDKN1B triplex structure. Our results verified p27 as a direct target of MIR100HG. Despite this, however, we cannot exclude the possibility that MIR100HG may also directly regulate p21 and cyclin D1 by additional mechanisms.

In summary, we found a novel mechanism for MIR100HG in TNBC. MIR100HG formed a triplex structure with p27. Through regulating the expression of p27, MIR100HG promoted cell proliferation in TNBC. Increased expression of MIR100HG was associated with poor prognosis. Thus, MIR100HG may be a potential biomarker of TNBC and may provide broader perspective for the treatment or cure of TNBC.

## Materials and methods

### Cell culture

MDA-MB-231 cells were cultured in DMEM/F12 medium with 10% fetal bovine serum (FBS) and 1% penicillin/streptomycin solution. BT549 cells were cultured in RPMI-1640 medium with 10% FBS and 1% penicillin/streptomycin solution. All cell lines were maintained in a cell culture incubator with 5% CO_2_ at 37 °C.

### RNA extraction and qPCR

Total RNA was isolated from cell lines using TRIzol reagent (Life Technologies). The cDNA was synthesized using a PrimeScript RT reagent kit (TaKaRa) according to the manufacturer’s instructions. After completing the reverse transcription of total RNA, qPCR was performed to detect the expressions of related genes using a SYBR Green PCR Master Mix (Applied Biosystems) on a QuanStudio 3 Applied Biosystems (Thermo Fisher Scientific). Primer sequences are listed in Supplementary Table S1.

### ShRNA-mediated knockdown

The shRNA targeting MIR100HG was inserted into the pLKO.1 cloning vector, and then co-transfected into 293T cells with vectors pMD2.G and psPAX2 at a ratio of 4:1:3 to produce a lentivirus. The packaged lentivirus was then used to infect MDA-MB-231 and BT549 cells, with sh-TRC (Addgene #10879) used as the control. After 72 h, the cells were collected for RNA and protein level detection. The sh-RNA sequences are listed in Supplementary Table S1.

### MIR100HG overexpression

The full-length sequence of MIR100HG was amplified and inserted into the pCDH vector (System Biosciences). The newly built constructs were transfected into 293T cells to produce a lentivirus. The packaged lentivirus was then used to infect MDA-MB-231 cells. We then generated MIR100HG stably expressed cells selected by 2 μg/ml puromycin for approximately 2 weeks starting at 4 days after virus infection. Cells were collected after 72 h to detect the efficiency of MIR100HG overexpression using qPCR.

### Cell proliferation assay

The infected MDA-MB-231 and BT549 cells with stable MIR100HG expression were seeded into 96-well plates at a density of 2 000 cells per well, then cultured for 0, 24, 48, and 72 h, respectively, in a cell culture incubator at 37 °C. Cells were then treated with 3-(4,5-dimethylthiazol-2-yl)-5-(3-carboxymethoxyphenyl)-2-(4-sulfophenyl)-2H tetrazolium, inner salt (MTS) (CellTiter 96® AQueous One Solution Cell Proliferation Assay) for 2 h. Optical density (OD) was measured with a plate reader (Biotek, model Synergy HTi) at 490 nm.

### Cell cycle analysis by flow cytometry

Cells were fixed in 70% ethanol at 4 °C for 48 h. Afterwards, the cells were washed in phosphate-buffered saline (PBS), stained with propidium iodide (PI), RNase A, and NP-40, and finally incubated at 37 °C for 30 min. Cell cycle distribution was detected by flow cytometry (BD, LSR Fortessa). Data were analyzed by FlowJo software.

### Bromodeoxyuridine (BrdU) staining

Cells were seeded in 24-well plates at an initial density of 1 × 10^5^ cells/well. After incubation with 10 μmol*/*L BrdU solution37 °C for 45 min, the cells were fixed with paraformaldehyde for 15 min at room temperature. The cells were thereafter washed once with PBS and treated with DNase for 15 min, then again washed with PBS and incubated with BrdU antibody for 8 h at 4 °C. Afterwards, the cells were incubated with secondary antibody for 1 h at room temperature. The nuclei were then stained with DAPI and at least five fields were selected for statistical analysis.

### Nuclear cytoplasmic separation

A PARIS™ kit was used to extract the cytoplasm and nucleus from MDA-MB-231 cells. RNA extracted from the cytoplasm and nucleus was used to detect the expression levels of *GAPDH*, *MALAT1*, and *MIR100HG* by qPCR. We used *GAPDH*, which is located in the cytoplasm, as a negative control and *MALAT1*, which is located in the nucleus, as a positive control.

### Western blot analysis

Cell were treated with lysis buffer for 30 min. Proteins were separated by sodium dodecyl sulfate polyacrylamide gel electrophoresis (SDS-PAGE) and transferred to polyvinylidenefluoride (PVDF) membranes (Millipore). The membranes were probed with specific antibodies against GAPDH (Abcam), cyclin D1 (Abcam), p21 (Abcam), and p27 (Abcam), then incubated for 1 h at room temperature with appropriate HRP-linked secondary antibodies (Sigma) and detected with chemiluminescent HRP substrate (Millipore).

### Mouse xenograft model

The MDA-MB-231 cells transduced with sh-TRC or sh-MIR100HG were resuspended (1 × 10^6^) in PBS/Matrigel mixture, and then injected orthotopically into 8-week-old female nude mice. Each experimental group contained six mice. All mice were sacrificed by cervical dislocation, with tumors then surgically collected and weighed. All experimental procedures and animal care and handling were performed per the protocols approved by the Ethics Committee of the Kunming Institute of Zoology, Chinese Academy of Sciences.

### RNA-seq data analysis

Barcodes were trimmed from RNA-seq raw reads, the quality of which was confirmed with FASTQC (v0.10.1). High-quality reads were mapped to the Genecode fasta file (GRCh37.primary_assembly.genome.fa) with STAR (STAR_2.4.2a). The Gencode annotation GTF file (gencode.v25lift37.annotation.gtf) was used to improve mapping accuracy. FeatureCounts(v1.4.6-p5) was used to assign sequence reads to exon features and then group exons into genes. Genes that possessed less than five raw reads in half the samples were deleted. The raw counts were normalized, and differentially expressed genes (DEGs) were determined using edgeR. Specifically, the quasi-likelihood method (glmLRT) in edgeR was used to determine DEGs. Log2FC > 2 or Log2FC < −2 and FDR < = 0.05 were used to isolate the final significant DEGs.

### Triplex structure prediction

We assembled all MIR100HG transcripts (in human genome hg38) into a lncRNA sequence and used the LongTarget program to predict the lncRNA sequence’s DNA-binding motif and binding sites in the CDKN1B region^41^. A clear triplex structure was generated at the CDKN1B promoter by TFO1 (best predicted DNA-binding motif) and TFO2 (second best predicted DNA-binding motif).

### In vitro triplex pull-down assay

Primers were designed to amplify the 5′UTR fragment (+ 141/ + 469) of p27 from the genome and the PCR product was digested with exonuclease I. Afterwards, 100 fmol of the PCR fragment was added and incubated with 1 pmol of biotin-labeled RNA in the triplex hybridization solutionat at 37 °C for 30 min. The RNA–DNA mixture was incubated with streptavidin beads at 37 °C for 40 min. Finally, the RNA-associated DNA was eluted with RNase A and proteinase K, analyzed by qPCR, and normalized to input DNA.

### Nuclei triplex capture assay

Nuclei triplex capture assay was performed followed the protocol from Postepska-Igielska A et al^38^. In brief, 3 × 10^6^ nuclei from MDA-MB-231 cells were incubated with 8 pmol biotinylated RNA in buffer (10 mM Tris-HCl [pH 7.5], 20 mM KCl, 10 mM MgCl_2_, and RNAs in) for 1 h. Nuclei were sonicated (10 cycles, 30 s ON and 30 s OFF) and spined at 10,000 RPM for 5 min. Then, supernatant was incubated with streptavidin beads for 40 min. RNA–DNA triplex complexes were washed three times with a buffer containing 150 mM KCl, 10 mM Tris-HCl (pH 7.5), 5 mM MgCl_2_, 0.5% NP-40, and RNasin and once with buffer containing 15 mM KCl, 10 mM Tris-HCl (pH 7.5), and 5 mM MgCl_2_. The RNA-associated DNA was eluted with 1% SDS, 50 mM Tris-HCl (pH 8.0), and 10 mM EDTA for 5 min at 65 °C and digested with RNase A (50 ng/ml, 30 min at 37 °C) and proteinase K (200 ng/ml, 15 min at 37 °C). The QPCR analysis was performed after DNA was purified with phenol-chloroform extraction and ethanol precipitation.

### Chromatin isolation by RNA purification (CHIRP)

First, 2 × 10^7^ cells were fixed with glutaraldehyde for 10 min and then ultrasonically broken into 200–500-bp DNA fragments. The biotin-labeled RNA was incubated with the DNA fragments at 37 °C for 4 h with shaking. We then added 100 μL of streptavidin beads to each tube. Finally, RNA-associated DNA was eluted with RNase A and proteinase K, analyzed by qPCR, and normalized to input DNA.

### TCGA data analysis

The raw sequence data were processed through the standard Illumina pipelines for base-calling and fastq file generation. Paired-end reads were mapped to the human genome primary assembly (GRCh37)^57^, and the Ensembl human gene annotation for GRCh37 genebuild was used to improve mapping accuracy with STAR software (STAR_2.4.2a)^58^. FeatureCounts (v1.4.6-p5)^59^ software was used to assign sequence reads to genes. Mitochondrial genes, ribosomal genes, and genes possessing fewer than five raw reads in half the samples were removed. Differential expression analysis was performed with the Bioconductor edgeR package 1.6^60^ using an over-dispersed Poisson model with a common dispersion parameter combined with the exact test. Significant genes were determined by an adjusted *P*-value of <0.05 based on the Benjamini-Hochberg multiple-testing correction and a log2-transformed fold change >2 or less than −2.

### Statistical analysis

All error bars indicate standard error mean (SEM). Student’s *t* test was used for two-sample comparisons. For multiple-sample comparisons, one-way or two-way analysis of variance (ANOVA) was performed, followed by Bonferroni’s multiple comparison test.

## Electronic supplementary material

Supplementary Table

Supplementary Figures

Supplementary figure legends
