# Supplementary material for: LncRNA MIR100HG promotes cell proliferation in triple-negative breast cancer through triplex formation with p27 loci
Source: Cell Death Dis. 2018 Jul 24;9(8):805. doi: 10.1038/s41419-018-0869-2 (PMC6057987; doi:10.1038/s41419-018-0869-2)
Supplement: Supplementary file 1 — Supplementary Table [file 41419_2018_869_MOESM1_ESM.doc]

**Table S1. Primers and shRNA sequences used in this paper**

| Primer | Sequence |
| --- | --- |
| GAPDH-F | GGGAAATTCAACGGCACAGT |
| GAPDH-R | AGATGGTGATGGGCTTCCC |
| MIR100HG-F | CCCAGTGCAAGGACAAAGA |
| MIR100HG-R | GCAGAGGAGGTGTCTTCAGG |
| CDKN1A-F | GGGTGGCTCACTCTTCTGGC |
| CDKN1A-R | TGGCCTTGCCCGGGCTTGTC |
| CDKN1B-F | AACGTGCGAGTGTCTAACGG |
| CDKN1B-R | CCCTCTAGGGGTTTGTGATTCT |
| BLID-F | TACACAGGATGGATAGAGCGAG |
| BLID-R | CTTTGTTGGAACCCAAGAGCG |
| UBASH3B-F | CCATGTCGGTGACCCCTTC |
| UBASH3B-R | GCTGTCCTCGCACATAAAGAA |
| SORL1-F | CAAGGTGTACGGACAGGTTAGT |
| SORL1-R | CCAATGCCAGGCTATCTCG |
| MALAT1-F | GTGTGCCAATGTTTCGTTTG |
| MALAT1-R | AGGAGAAAGTGCCATGGTTG |
| p27 TTS-F | CTGGTCCCCTCTCCTCTCC |
| p27 TTS-R | CCTCGGAGCCAAAAGACACA |
| shRNA oligo |  |
| sh-MIR100HG-1F | CCGGTTCCTCTGTTTGTACTTAAATCTCGAGATTTAAGTACAAACAGAGGAATTTTTG |
| sh-MIR100HG-1R | AATTCAAAAATTCCTCTGTTTGTACTTAAATCTCGAGATTTAAGTACAAACAGAGGAA |
| sh-MIR1000HG-2F | CCGGGAGAGTGACCTACAGAATAAACTCGAGTTTATTCTGTAGGTCACTCTCTTTTTG |
| sh-MIR1000HG-2R | AATTCAAAAAGAGAGTGACCTACAGAATAAACTCGAGTTTATTCTGTAGGTCACTCTC |
