# Supplementary figures and images for: LncRNA MIR100HG promotes cell proliferation in triple-negative breast cancer through triplex formation with p27 loci
Source: Cell Death Dis. 2018 Jul 24;9(8):805. doi: 10.1038/s41419-018-0869-2 (PMC6057987; doi:10.1038/s41419-018-0869-2)

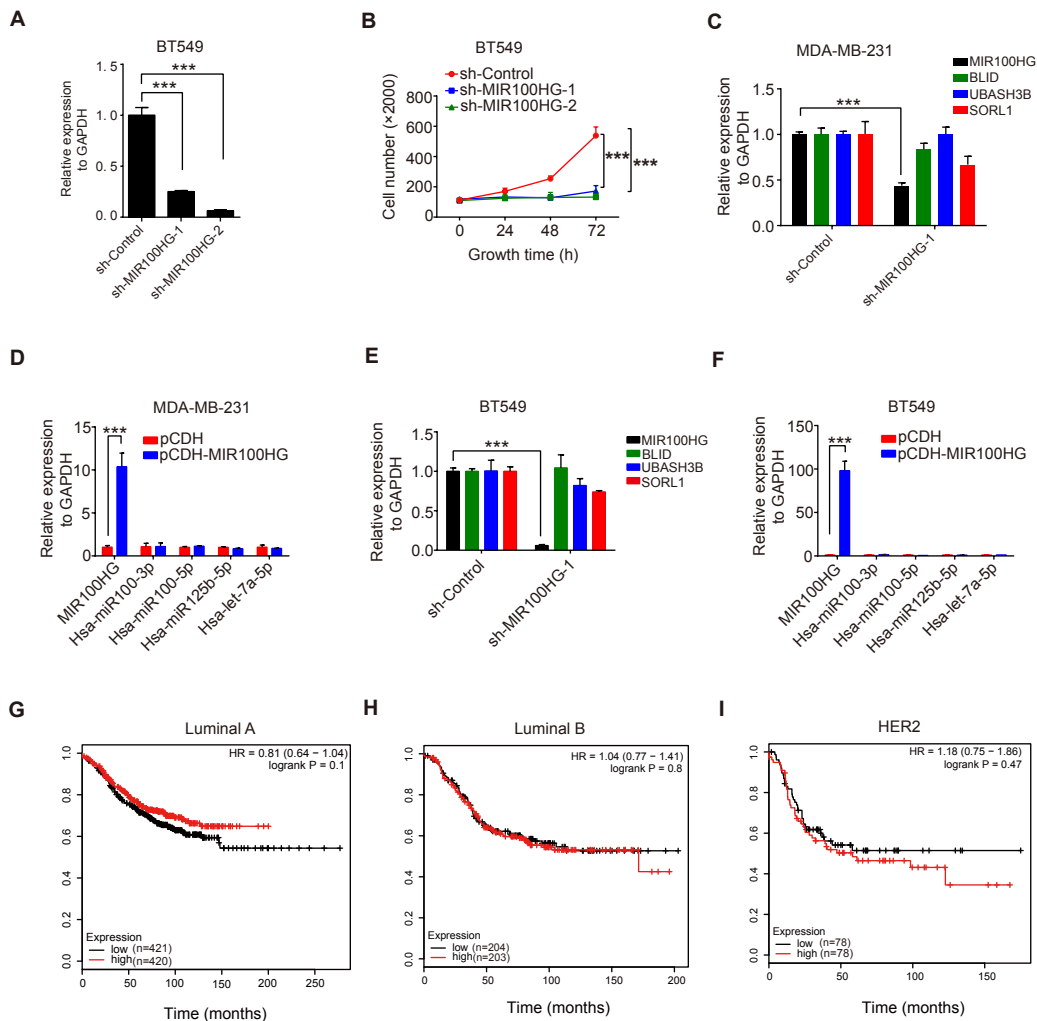

A

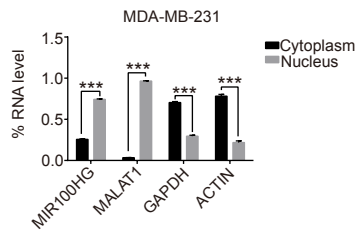

B

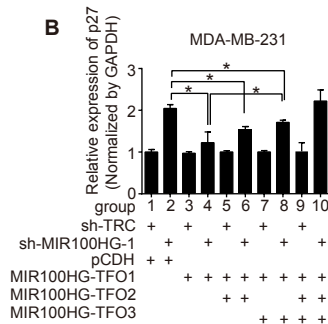

C

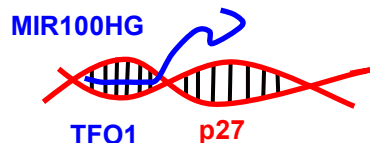

D

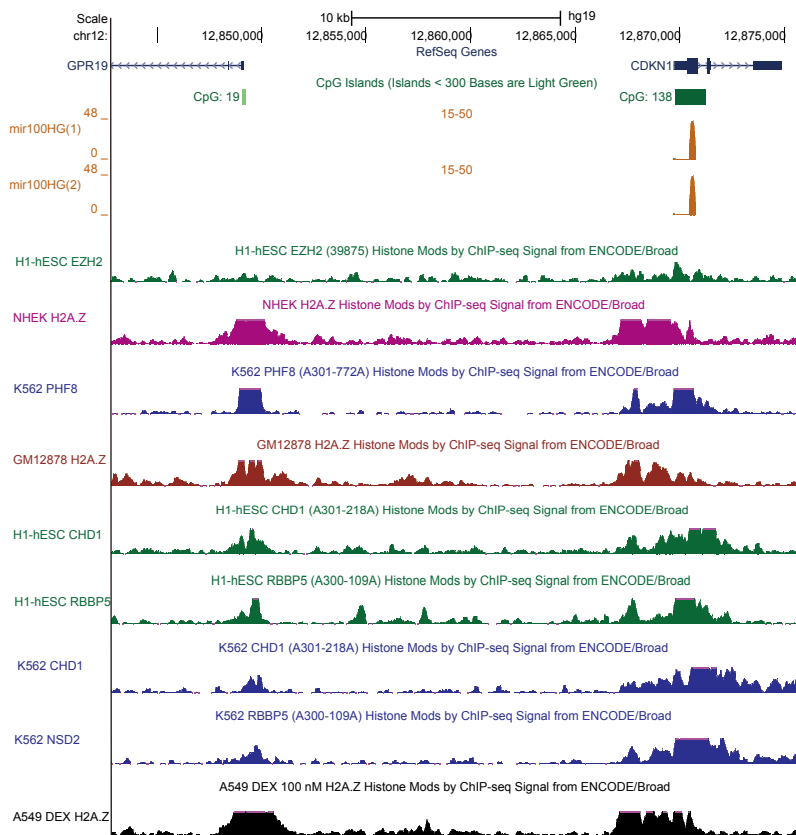

Supplement: Supplementary file 2 — Supplementary Figures [file 41419_2018_869_MOESM2_ESM.pdf]
