# Supplementary material for: LncRNA MIR100HG promotes cell proliferation in triple-negative breast cancer through triplex formation with p27 loci
Source: Cell Death Dis. 2018 Jul 24;9(8):805. doi: 10.1038/s41419-018-0869-2 (PMC6057987; doi:10.1038/s41419-018-0869-2)
Supplement: Supplementary file 3 — Supplementary figure legends [file 41419_2018_869_MOESM3_ESM.docx]

**Supplementary figure legends**

Figure S1. (A) Knockdown efficiencies of two shRNAs for MIR100HG in BT549 cells. Transcript levels of MIR100HG were normalized to GAPDH expression. (B) MTS assay of proliferation of sh-MIR100HG-transfected BT549 cells. (C, E) qPCR analysis of expression levels of neighboring genes *BLID*, *UBASH3B*, and *SORL1* after MIR100HG downregulation in MDA-MB-231 (C) and BT549 cells (E). (D, F) qPCR analysis of expression levels of has-miR100-3p, has-miR100-5p, has-miR125b-5p, and has-let-7a-5p after overexpression of MIR100HG in MDA-MB-231 (D) and BT549 cells (F). (G-I) Kaplan-Meier curves for overall survival rate of 841, 407, and 156 patients with luminal A, luminal B, and HER2, respectively, by MIR100HG expression in tumors. ****P* < 0.001 by one-way ANOVA.

Figure S2. (A) qPCR detection of MIR100HG levels in cellular fractions from MDA-MB-231 cells. MALAT1 and GAPDH were applied as controls for the nucleus and cytoplasm, respectively. (B) P27 levels by MIR100HG knockdown with/without overexpression of pCDH-MIR100HG-TFO1, pCDH-MIR100HG-TFO2, and pCDH-MIR100HG-TFO3. (C) Working model illustrating the RNA-DNA triplex structures formed by MIR100HG, through the TFO1 sequence, and p27 gene loci. (D) TFOs in MIR100HG matched the triplex target DNA sites (TTSs) of human CDKN1B. **P* < 0.05, ****P* < 0.001 by one-way ANOVA.
